# Immune responses in the treatment of drug-sensitive pulmonary tuberculosis with phenylbutyrate and vitamin D_3_ as host directed therapy

**DOI:** 10.1186/s12879-018-3203-9

**Published:** 2018-07-04

**Authors:** Rokeya Sultana Rekha, Akhirunnesa Mily, Tajnin Sultana, Ahsanul Haq, Sultan Ahmed, S. M. Mostafa Kamal, Annemarie van Schadewijk, Pieter S. Hiemstra, Gudmundur H. Gudmundsson, Birgitta Agerberth, Rubhana Raqib

**Affiliations:** 1 Infectious Diseases Division, icddr,b, 68 Shaheed Tajuddin Ahmed Sarani, Dhaka, 1212 Bangladesh; 2 Department of Laboratory Medicine, Clinical Microbiology, Karolinska Institutet, Karolinska University Hospital, Stockholm, Sweden; 3 National Institute of the Diseases of the Chest and Hospital, Mohakhali, Dhaka, Bangladesh; 4 Department of Pulmonology, Leiden University Medical Centre, Leiden, the Netherlands; 5 Biomedical Center, University of Iceland, Reykjavik, Iceland

**Keywords:** *Mycobacterium tuberculosis*, Cytokines, Chemokines, Endoplasmic reticulum stress, Human beta-defensin-1 (HBD1)

## Abstract

**Background:**

We have previously shown that 8 weeks’ treatment with phenylbutyrate (PBA) (500mgx2/day) with or without vitamin D_3_ (vitD_3_) (5000 IU/day) as host-directed therapy (HDT) accelerated clinical recovery, sputum culture conversion and increased expression of cathelicidin LL-37 by immune cells in a randomized, placebo-controlled trial in adults with pulmonary tuberculosis (TB). In this study we further aimed to examine whether HDT with PBA and vitD_3_ promoted clinically beneficial immunomodulation to improve treatment outcomes in TB patients.

**Methods:**

Cytokine concentration was measured in supernatants of peripheral blood mononuclear cells (PBMC) from patients (*n* = 31/group). Endoplasmic reticulum stress-related genes (*GADD34* and *XBP1spl*) and human beta-defensin-1 (HBD1) gene expression were studied in monocyte-derived-macrophages (MDM) (*n* = 18/group) from PBMC of patients. Autophagy in MDM (*n* = 6/group) was evaluated using LC3 expression by confocal microscopy.

**Results:**

A significant decline in the concentration of cytokines/chemokines was noted from week 0 to 8 in the PBA-group [TNF-α (β = − 0.34, 95% CI = − 0.68, − 0.003; *p* = 0.04), CCL11 (β = − 0.19, 95% CI = − 0.36, − 0.03; *p* = 0.02) and CCL5 (β = − 0.08, 95% CI = − 0.16, 0.002; *p* = 0.05)] and vitD_3_-group [(CCL11 (β = − 0.17, 95% CI = − 0.34, − 0.001; p = 0.04), CXCL10 (β = − 0.38, 95% CI = − 0.77, 0.003; p = 0.05) and PDGF-β (β = − 0.16, 95% CI = − 0.31, 0.002; p = 0.05)] compared to placebo. Both PBA- and vitD_3_-groups showed a decline in *XBP1spl* mRNA on week 8 (*p* < 0.03). All treatment groups demonstrated increased LC3 expression in MDM compared to placebo over time (*p* < 0.037).

**Conclusion:**

The use of PBA and vitD_3_ as adjunct therapy to standard TB treatment promoted favorable immunomodulation to improve treatment outcomes.

**Trials registration:**

This trial was retrospectively registered in clinicaltrials.gov, under identifier NCT01580007.

**Electronic supplementary material:**

The online version of this article (10.1186/s12879-018-3203-9) contains supplementary material, which is available to authorized users.

## Background

Tuberculosis remains among the top 10 leading causes of death with global estimates of 10.4 million new cases and 1.4 million deaths in 2015 [1]. Control or exacerbation of TB is dependent on host immune responses generated to combat *Mycobacterium tuberculosis* (*Mtb*) infection [2–4]. Several studies have unravelled immunological pathways that influence the outcome of *Mtb* infection which include cytokine-mediated signalling among T cells, macrophages and neutrophils, and phagocytes-mediated antimicrobial processes [4–9]. Studying cytokine profiles in TB patients has demonstrated its potential for use in diagnostic purposes, monitoring of treatment efficacy and development of novel treatment strategies [10–14]. Autophagy in macrophages and intracellular lysosomal degradation are important for killing of pathogens although *Mtb* has evolved to escape elimination by blocking phagosomal acidification and phagosome-lysosomal fusion [15–17]. Endoplasmic-reticulum (ER) stress response is triggered by mycobacterial infection and plays a critical role in the pathogenesis of TB [18].

The rise in antibiotic resistance among *Mtb* in the last decade rekindled attention towards alternative chemotherapies. Host-directed therapies (HDT) have emerged as a promising avenue for adjunctive treatment with the aim to modulate immune responses against the pathogen by targeting clinically relevant host biological pathways. This strategy would be beneficial in reducing the course of antibiotic treatment, preventing the spread of drug-resistant *Mtb*, and reducing inflammation in the lung (reviewed in [19, 20]). Phenylbutyrate (PBA) is a licensed drug indicated for the management of urea cycle disorders [21]. Vitamin D_3_ (vitD_3_), a dietary supplement, has diverse immune-modulatory properties. Our group has shown that PBA and vitD_3_ have a strong synergistic effect on induction of antimicrobial peptides (AMPs) in lung epithelial cell lines, macrophages, and in healthy human immune cells [22–24]. In a randomized clinical trial, we have shown that PBA alone or combined with vitD_3_ is a promising candidate for HDT in the treatment of drug sensitive pulmonary TB by speeding up clinical recovery [25]. Treatment with vitD_3_ or the combination with PBA accelerated sputum culture conversion and enhanced expression of LL-37, the human AMP of the cathelicidin family, by immune cells. Moreover, PBA adjunctive therapy increased macrophage-mediated killing of *Mtb* ex vivo compared to placebo. Our in vitro study further showed that PBA can induce autophagy in a LL-37 dependent pathway and promotes intracellular killing of *Mtb* in human macrophages [23]. PBA is known to reduce ER stress in cells and thereby reduce inflammatory responses [26, 27]. We hypothesized that the beneficial effects imparted by HDT of PBA and vitD_3_ in TB patients may be mediated through regulating expression of cytokines, chemokines and AMPs by immune cells, augmentation of autophagic responses of macrophages and reduction of chronic ER stress. Thus, in a sub-group of TB patients, we evaluated the effect of HDT on dynamics of cytokines and chemokines in culture supernatants of PBMC, HBD1 and ER stress genes and expression of LC3, an autophagy marker, in macrophages from TB patients in response to the disease and clinical improvement.

## Methods

### Patients, study design and interventions

For this study, we used materials collected during our previously published clinical trial [25]. Briefly, 288 adult patients with newly diagnosed sputum smear-positive TB (18–55 years of age) were recruited from the National Institute of the Diseases of the Chest and Hospital (NIDCH) in Dhaka, Bangladesh. The study was approved by the Research and Ethical Review Committees at icddr,b, an international health research institute based in Bangladesh. The study was a double-blind, placebo-controlled trial in which patients were randomized to the following adjunct therapy arms (72 × 4) receiving oral doses of either: (1) placebo PBA and placebo vitD_3_; or (2) 500 mg twice daily of PBA and placebo vitD_3_; or (3) placebo PBA and 5000 IU of vitD_3_ (Cholecalciferol) once daily; or (4) PBA combined with vitD_3_ (PBA + vitD_3_). In parallel, standard care of directly observed therapy short-course (DOTS) regimen consisting of isoniazid (INH), rifampicin (RIF), pyrazinamide, and ethambutol was given to all patients for 2 months followed by INH and RIF for 4 months. Clinical evaluation, sputum microscopy, sputum culture and chest radiography were performed [25]. A total of 249 patients (modified intention-to-treat) completed the week 12 follow-up and 219 completed week 24 follow-up visit.

In the published TB trial, clinical assessments were performed by the study doctor and were used to calculate numerical clinical scores which was defined as a TB score [25]. The TB score is an evaluation tool developed by clinicians to quantify/determine changes in clinical symptoms of the TB patients in an impartial and objective manner. The TB score allocated points for self-reported symptoms (cough, shortness of breath/dyspnea, chest pain, haemoptysis, anorexia), and clinical signs assessed by study doctors (fever, anemia (< 11 g/dl), tachycardia, auscultatory findings). The TB score was determined at week 0–4, 6, 8, 10, 12 and 24.

For the present study, a sub-sample of 127 patients (32/group) was selected for studying cytokine and chemokine responses of PBMC for which adequate volume and complete set of PBMC culture supernatants at 2 time points (week 0 and 8) were available. In the vitD_3_ group, a complete set of 31 patients were only available. ER stress (18/group) and HBD1 (18/group) genes were studied using cDNA synthesized from monocyte-derived-macrophages (MDM) of the TB patients at week 0, 4 and 8. For studying autophagy marker, MDM from 24 patients (6/group) were selected with adequate number of cells available at 4 time points (week 0, 4, 8 and 12).

### Samples and cell culture supernatant

**Fig. 1:**
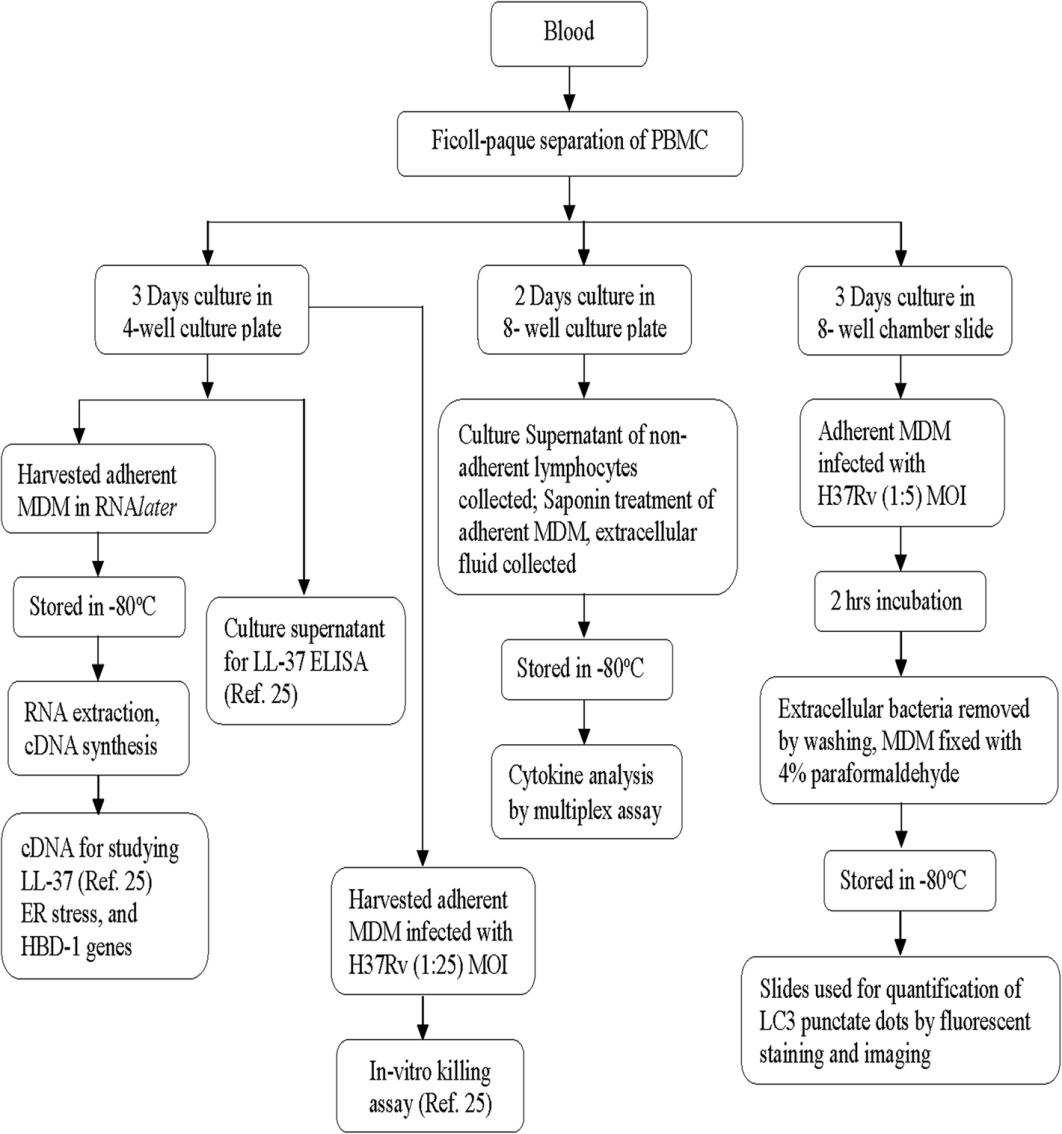
The flow chart illustrates collection, processing and storage of different cell types from freshly isolated peripheral blood mononuclear cells (PBMC), the time periods of cell culture and the various signature immune markers evaluated in these patients

Peripheral blood mononuclear cells (PBMC) and plasma were separated from whole blood by Ficoll-PaqueTM PLUS (GE Healthcare, Uppsala, Sweden) density gradient centrifugation. PBMC were used directly following isolation and were not stored in liquid nitrogen for future use. After washing, PBMCs were suspended in culture medium and cultured for 2 days. Intracellular fluid was collected after adding 0.1% saponin (Sigma-Aldrich, Steinheim, Germany) and stored in ultra-low freezer for analyses of cytokines (Fig. 1). Forty-eight hours of incubation of PBMC without any stimulation allows partial maturation of monocytes in the mixed cell culture (T and B lymphocytes, NK cells, plasma cells and monocytes). There is spontaneous and active release of cytokines or antibodies directly from the immune cells into the culture fluid (extracellular cytokines); reversible permeabilization of PBMC by saponin additionally allows excretion of intra-cellular cytokines into the supernatant.

Five million PBMC were plated in 4-well culture plates (NUNC, Roskilde, Denmark) for three days, after removing nonadherant cells the adherent MDMs were harvested using a cell scraper, treated with RNA*later* (Qiagen GmbH, Hilden, Germany). Total RNA was isolated from MDM using the RNeasy Mini Kit (Qiagen GmbH). mRNA was reverse-transcribed and corresponding cDNA was synthesized using the SuperScript III First-Strand Synthesis System (Invitrogen Life Technologies, CA) with CFX96 real-time PCR detection System (Bio-Rad, Hercules, CA, USA). The cDNA was used for analysis of ER stress-related genes and HBD1 (Fig. 1).

### Cytokines and chemokines

Multiplex kits (Bio-Rad) were used to analyze cytokines and chemokines in the cell supernatants of PBMC following manufacturer’s instructions in the Bioplex 200 system (Bio-Rad). Two types of kits were used, (i) the 27-plex kit contained a panel of 27 analytes: IL-1β, IL-1ra, IL-2, IL-4, IL-5, IL-6, IL-7, IL-8, IL-9, IL-10, IL-12(p70), IL-13, IL-15, IL-17, CCL11 (eotaxin), FGF basic, G-CSF, GM-CSF, IFN-γ, CXCL10 (IP-10), CCL2 (MCP-1), CCL3 (MIP-1α), CCL4 (MIP-1β), PDGF-BB, CCL5 (RANTES), TNF-α, and VEGF. (ii) The 17-plex kit contained: IL-1β, IL-2, IL-4, IL-5, IL-6, IL-7, IL-8, IL-10, IL-12(p70), IL-13, IL-17, G-CSF, GM-CSF, IFN-γ, CCL2, CCL3 and TNF-α. For 75 patients 27-plex kit was used (19/group; 18/vitD_3_ group), while for the rest of 52 patients (13/group) the 17-plex kit was utilized, therefore results for 8 analytes were not available for 52 patients.

### Autophagy marker in macrophages

Autophagy is a physiological process; it is applied by macrophages to control growth or elimination of intracellular pathogens. LC3 is a classical marker and most widely used for assessing the autophagy process LC3-I is the cytosolic form of this protein. Upon activation of autophagy, LC3-I is subsequently conjugated with phosphatidylethanolamine (PE) to generate LC3-II by a ubiquitination-like enzymatic reaction. In contrast to the cytoplasmic localization of LC3-I, LC3-II associates with both the outer and inner membranes of the autophagosome. This LC3-II protein looks like dots and is known as ‘Puncta’. In a previous study, we have shown that treatment of MDM with PBA increased the expression of LC3 with simultaneous decrease in p62 protein expression compared to untreated MDM [23]. Due to the availability of limited number of cells from patients at each time point, simultaneous staining of both LC3 and p62 was not feasible.

Freshly isolated PBMCs from TB patients [25] (above) were cultured in culture medium for 3 days in 8-well chamber slides (Nunc® Lab-Tek® Chamber Slide™ system). After removal of non-adherent cells, adherent MDM were infected with virulent *Mtb* strain, H37Rv (Tuberculosis Research Center, Chennai, India) at a multiplicity of infection (MOI) of 1:5 (1 macrophage to 5 bacilli (*Mtb*)) in culture media for 2 h without antibiotics in the BSL-3 facility. After washing to remove the extracellular bacteria, the infected cells were fixed with 4% paraformaldehyde (Sigma-Aldrich) in phosphate buffered saline and stored in − 80 °C until shipped to the Laboratory in Karolinska Institute. The frozen slides were brought to room temperature, and staining was performed as mentioned earlier [23]. Quantification of autophagy was performed based on the percentage of the cells with LC3 punctate dots by ImageJ software (National Institutes of Health, US).

### Endoplasmic reticulum stress in MDM

As markers of ER stress, growth arrest and DNA damage inducible protein 34 (*GADD34*) and spliced X-box binding protein-1 (*XBP1spl*) mRNA were assessed in MDM from TB patients (Fig. 1). The qPCR method was performed as described previously [28] using the primer pairs as follows: *GADD34* forward primer 5’-ATGTATGGTGAGCGAGAGGC-3′, reverse primer 5’-GCAGTGTCCTTATCAGAAGGC-3′; *XBP1spl* forward primer 5’-TGCTGAGTCCGCA-GCAGGTG-3′, reverse primer 5’-GCTGGCAGGCTCTGGGGAAG-3′. Relative mRNA concentration of the reference gene ATP5B, (GeNorm, PrimerDesign Ltd., Southampton, UK) was used to calculate normalized expression. Each assay was run on a CFX96 (Bio-Rad) in triplicates and arbitrary mRNA concentrations were calculated by the Bio-Rad software, using the relative standard curve method.

### Human beta-defensin-1 (HBD1) in MDM

Expression of the gene encoding human β-defensin-1 was measured by qPCR using CFX96 (Bio-Rad) (Fig. 1). The following primer sequences were used for quantification of HBD1. Sense primer: 5′-ATGGCCTCA-GGTGGTAACTTTC-3′; antisense primer: 5′-CACTTGGCCTTCCCTCTGT-AAC-3′. Relative quantification method was used to the housekeeping RNA18S/18S rRNA (18S rRNA–housekeeping gene kit, Applied Biosystems, Foster. City, USA). Results were expressed as relative gene expression and the 2^–ΔΔC^T method or fold changes. ΔCt values were calculated by subtracting 18 s rRNA Ct values from HBD1 Ct values of the same sample. Thereafter, ΔΔCt was obtained by subtracting average placebo week 0 ΔCt value from all ΔCt values. Finally, HBD1 relative gene expression was calculated by the formula, 2^-ΔΔCt^ [29].

### Statistical analysis

Distribution of different adjunct treatments (Placebo, PBA, vitD_3_ and PBA + vitD_3_) were calculated based on demographic characteristics (age, gender, history of contact, body weight, vitamin D status). Cytokine and chemokine data was log transformed due to non-normal distribution, and a simple interpretation of the beta-coefficient (average changes in outcome (concentration of cytokines, chemokines, number (%) of autophagy marker LC3 etc.) associated with change in treatment group with respect to placebo). Cytokine and chemokine responses of each of the 127 patients were studied at 2 time points, at week 0 and week 8. Wilcoxon Signed Ranks test was performed to evaluate the changes in cytokine and chemokine concentrations within each treatment group. To assess the treatment effects on changes in outcome variables (cytokine/chemokine concentrations and autophagy marker LC3^+^ cell counts) over time, generalized estimating equation (GEE) model was performed considering exchangeable correlation matrix. Each cytokine, chemokine and LC3^+^ cell counts (autophagy marker) was individually analyzed using the GEE model allowing repeated measures per patient to reduce longitudinal multicollinearity. The treatment effects were adjusted by covariates that influence the model by at least 5% (age, sex, duration of symptoms, history of contact with active TB cases and time at week 0 and week 8 (week 4 and 12 were considered where applicable)). The interaction between the treatment groups and time were also minimized. An ANCOVA model was applied to see the mean difference of *gadd34*, *xbp1spl* and HBD1 among different treatment groups. In both cases of GEE and ANCOVA, a *p*-value of < 0.05 was considered significant. All the data were analyzed by using the Statistical package for the Social Science (SPSS) for Windows (version 20; Armonk, NY: IBM SPSS corp.; 2011) and Stata/IC, version13 (Stata Corp, Texas, USA).

## Results

### Patients and clinical scores

Of total 249 patients, samples from 127 patients were included in the present study where mean age of the patients was 27.25 ± 8.37 years and female male ratio was 1:1.86 (35:65). The patient groups in the present study did not differ significantly in demographic and baseline characteristics amongst themselves (Table 1) or from the primary patient cohort who were not included in the present study (Additional file 1: Table S1).

**Table 1:** Demographic characteristics of the study participants

Characteristics	Placebo (*n* = 32)	PBA (*n* = 32)	vitD_3_ (*n* = 31)	PBA + vitD_3_ (*n* = 32)
Age, years	27.38 ± 8.65	27.72 ± 7.25	25.42 ± 8.15	27.16 ± 8.38
Males	20 (62.50%)	22 (25.90%)	21 (67.70%)	22 (68.80%)
History of contacts	10 (31.30%)	11 (34.40%)	6 (19.40%)	8 (25.00%)
BCG given	22 (68.8%)	19 (59.4%)	26 (83.90%)	23 (71.90%)
Weight, kg	44.75 ± 8.20	45.74 ± 8.91	42.91 ± 8.70	44.00 ± 7.89
Duration of illness, days	48.91 ± 25.42	50.63 ± 21.80	58.39 ± 26.81	52.47 ± 27.58
Vitamin D status				
Deficient, < 30 nmol/L	18 (56%)	23 (71%)	21 (67%)	16 (50%)
Insufficient, 30–50 nmol/L	10 (31.30%)	6 (18.8%)	5 (16.10%)	12 (37.50%)
Sufficient, > 50 nmol/L	4 (12.50%)	3 (9.4%)	5 (16.10%)	4 (12.50%)
Baseline clinical score (25)	6.47 ± 0.86	6.45 ± 1.09	6.27 ± 1.01	6.39 ± 0.96
Baseline sputum smear				
< 3 acid-fast bacilli	17 (43.10%)	17 (43.10%)	16 (51.60%)	18 (56.20%)
> 3 acid-fast bacilli	15 (46.90%)	15 (46.90%)	15 (48.40%)	14 (43.80%)

Data is presented as mean ± standard deviation or number with percentage in parentheses

Abbreviations: BCG Bacillus Calmette–Guérin

Longitudinal change in TB scores as analyzed by GEE showed a significant decline over time (week 0, 4, 8 and 12) in the PBA group compared to placebo (Additional file 1: Table S2) in the present cohort (*n* = 127). A similar reduction in TB scores was obtained in the PBA group compared to placebo in the parent cohort of 249 patients [25]. Significantly higher percentages of patients in the PBA + vitD_3_ and vitD_3_ groups became sputum culture negative at week 4 compared to placebo though no difference was obtained with sputum smear conversion.

### Cytokines and chemokines

Concentrations of cytokines IL-2, IL-5, IL-13 and IL-15 in PBMC supernatants were low or below detection limit in most patients. The other cytokines and chemokines showed a wide range of concentrations in all the supernatants measured. Concentrations of TNF-α, IL-17 (*p* = 0.05) and CCL11 (eotaxin) (*p* = 0.01) declined significantly from week 0 to week 8 in the PBA group. In the PBA + vitD_3_ group, FGF-basic and PDGF-β reduced in concentrations after 8 weeks from week 0 (*p* = 0.01) (Additional file 1: Table S3). Other groups did not show any significant reduction in cytokine/chemokine concentrations with time.

Analysis by GEE method exhibited a significant decline in TNF-α (β = − 0.34, 95% CI = − 0.68, − 0.003, *p* = 0.04) concentrations from week 0 to week-8 in the PBA group compared to placebo (Table 2). A significant reduction in the concentrations of the chemokines CCL11 (β = − 0.19, 95% CI = − 0.36, − 0.03, *p* = 0.02) and CCL5 (RANTES) (β = − 0.08, 95% CI = − 0.16, 0.002, *p* = 0.05) was observed in the PBA group, while CCL11 (β = − 0.17, 95% CI = − 0.34, − 0.001, p = 0.04), CXCL10 (IP-10) (β = − 0.38, 95% CI = − 0.77, 0.003, *p* = 0.05) and PDGF-β (β = − 0.16, 95% CI = − 0.31, 0.002, *p* = 0.05) declined significantly in the vitD_3_ group (Table 2). However, no differences in chemokine concentrations were noted in the PBA + vitD_3_ group compared to placebo. The results demonstrated that 8 weeks’ adjunct therapy with oral PBA or vitD_3_ effectively decreased expression of mononuclear cell-derived inflammatory cytokines and chemokines thereby reflecting reduced inflammatory responses in TB patients.

**Table 2:** Longitudinal change (week 0 and 8) in inflammatory cytokines in treatment groups compared to placebo

	Crude		Adjusted^a^	
	β(95% CI)	*p*-value	β(95% CI)	*p*-value
TNF-α				
PBA (n = 32)	− 0.17 (− 0.42, 0.09)	0.20	− 0.34 (− 0.68, − 0.003)	0.04
vitD_3_ (n = 31)	− 0.16 (− 0.41, 0.10)	0.23	− 0.22 (− 0.57, 0.13)	0.22
PBA + vitD_3_ (n = 32)	−0.13 (− 0.38, 0.13)	0.32	− 0.03 (− 0.37, 0.31)	0.85
IL-8				
PBA (n = 32)	−0.29 (− 0.68, 0.09)	0.13	− 0.33 (− 0.71, 0.05)	0.08
vitD_3_ (n = 31)	− 0.11 (− 0.50, 0.28)	0.57	−0.09 (− 0.49, 0.30)	0.63
PBA + vitD_3_ (n = 32)	−0.17 (− 0.55, 0.22)	0.39	−0.18 (− 0.55, 0.20)	0.36
GM-CSF				
PBA (n = 32)	−0.13 (− 0.38, 0.11)	0.28	− 0.27 (− 0.56, 0.01)	0.06
vitD_3_ (n = 31)	− 0.19 (− 0.44, 0.05)	0.12	−0.19 (− 0.49, 0.10)	0.20
PBA + vitD_3_ (n = 32)	0.08 (−0.17, 0.32)	0.54	0.06 (−0.23, 0.35)	0.69
IL-4				
PBA (n = 32)	−0.10 (− 0.23, 0.03)	0.14	− 0.14 (− 0.31, 0.01)	0.08
vitD_3_ (n = 31)	− 0.08 (− 0.21, 0.06)	0.26	−0.11 (− 0.27, 0.06)	0.20
PBA + vitD_3_ (n = 32)	−0.06 (− 0.20, 0.07)	0.35	−0.05 (− 0.22, 0.11)	0.52
CCL11 (eotaxin)				
PBA (n = 20)	−0.03 (− 0.17, 0.10)	0.63	− 0.19 (− 0.36, − 0.03)	0.02
vitD_3_ (*n* = 17)	−0.11 (− 0.26, 0.03)	0.11	−0.17 (− 0.34, − 0.001)	0.04
PBA + vitD_3_ (*n* = 19)	−0.04 (− 0.18, 0.10)	0.59	−0.07 (− 0.23, 0.10)	0.44
CCL5 (RANTES)				
PBA (*n* = 20)	−0.07 (− 0.13, − 0.001)	0.04	−0.08 (− 0.16, 0.002)	0.05
vitD_3_ (*n* = 17)	−0.05 (− 0.12, 0.01)	0.11	−0.06 (− 0.15, 0.02)	0.14
PBA + vitD_3_ (*n* = 19)	−0.04 (− 0.10, 0.03)	0.27	−0.06 (− 0.14, 0.03)	0.18
CXCL10 (IP-10)				
PBA (n = 20)	−0.11 (− 0.41, 0.18)	0.45	− 0.13 (− 0.51, 0.24)	0.47
vitD_3_ (n = 17)	− 0.25 (− 0.57, 0.06)	0.10	−0.38 (− 0.77, 0.003)	0.05
PBA + vitD_3_ (n = 19)	−0.15 (− 0.46, 0.15)	0.32	−0.16 (− 0.54, 0.21)	0.39
PDGF-BB				
PBA (n = 20)	−0.03 (− 0.18, 0.12)	0.71	0.01 (− 0.15, 0.16)	0.92
vitD_3_ (n = 17)	−0.16 (− 0.32, − 0.003)	0.04	−0.16 (− 0.31, 0.002)	0.05
PBA + vitD_3_ (n = 19)	−0.12 (− 0.28, 0.03)	0.10	−0.12 (− 0.27, 0.03)	0.12

Data is presented as beta coefficient with 95% confidence intervals in parentheses

*Abbreviations*: *CI* confidence interval, *PBA* phenylbutyrate, *vitD*_*3*_ vitamin D_3_, *TNF-α* tumor necrosis factor-alpha, *IL* interleukin, *GM-CSF* granulocyte-macrophage-colony stimulating factor, *PDGF-BB* platelet-derived growth factor-BB

^a^Adjusted for age, sex, duration of treatment, history of contact with active TB cases.

Statistical analysis was performed using generalized estimating equation (GEE) model. *P*-value of < 0.05 is significant

### Autophagy marker in ex vivo infected macrophages

**Fig. 2:**
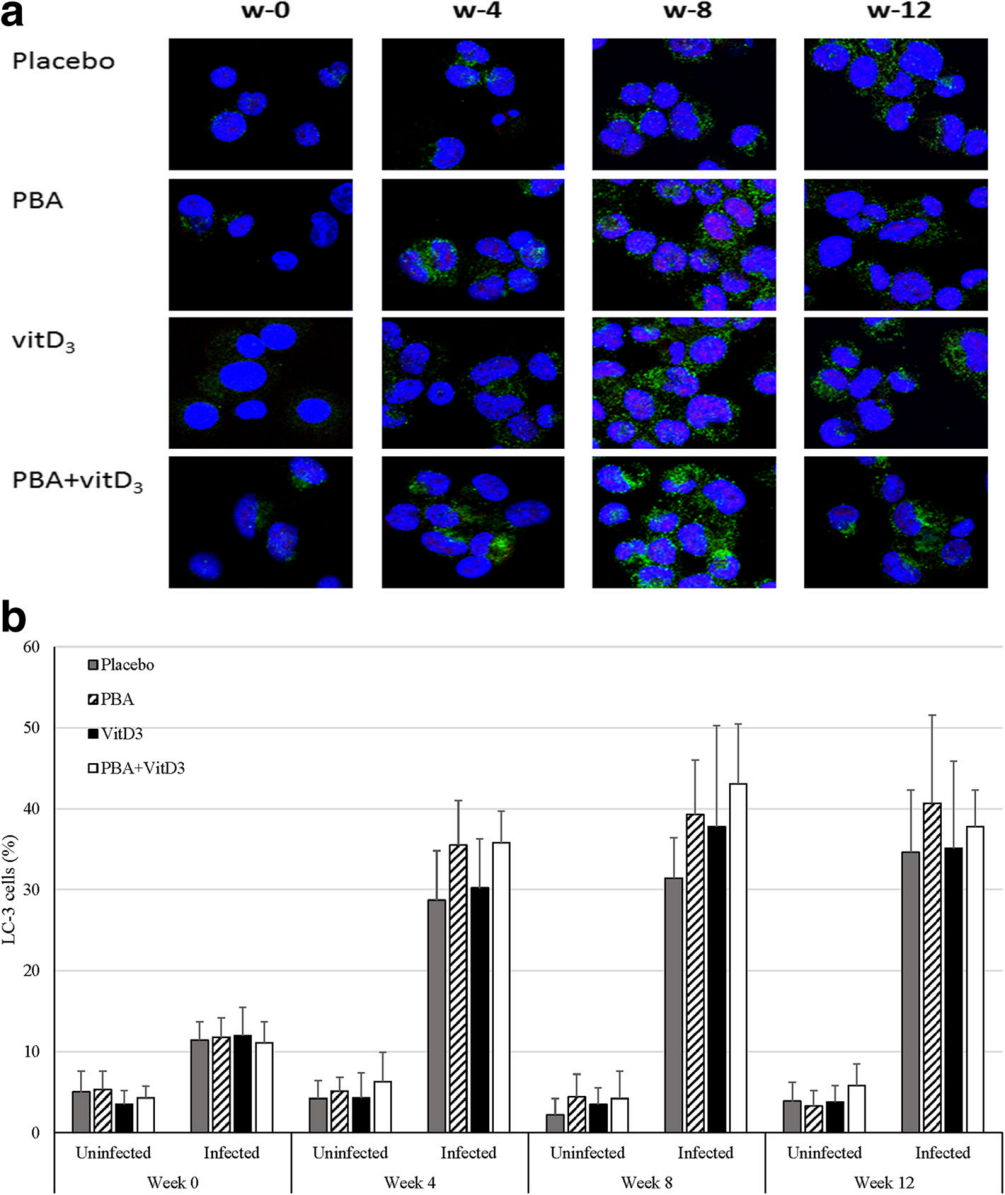
**a** Expression of LC3^+^ macrophages in TB patients after adjunct therapy with PBA and/or vitD_3_ and simultaneously under treatment with anti-TB drugs. MDMs derived from the patients were infected with the virulent strain of Mycobacterium tuberculosis H37Rv for 2 h. The cells were fixed and stained with DAPI to visualize the nuclei (blue), and with anti-LC3, followed by the addition of Alexafluor 488-conjugated goat anti-mouse IgG (green color). One representative immunofluorescence image out of 6 independent replicates are shown; scale bars: 10 μm. **b** Percentage of LC3 expressing cells among uninfected and *Mtb*-infected monocyte-derived-macrophages (MDM) in the four treatment arms

Confocal microscopic analysis revealed the activation of autophagy process in macrophages measured by the presence of LC3 positive puncta in MDM. LC3 puncta structures in ex vivo infected MDM showed highest percentage of cells with LC3-positive puncta in PBA + vitD_3_ group at week 8 (Fig. 2a).

**Fig. 3:**
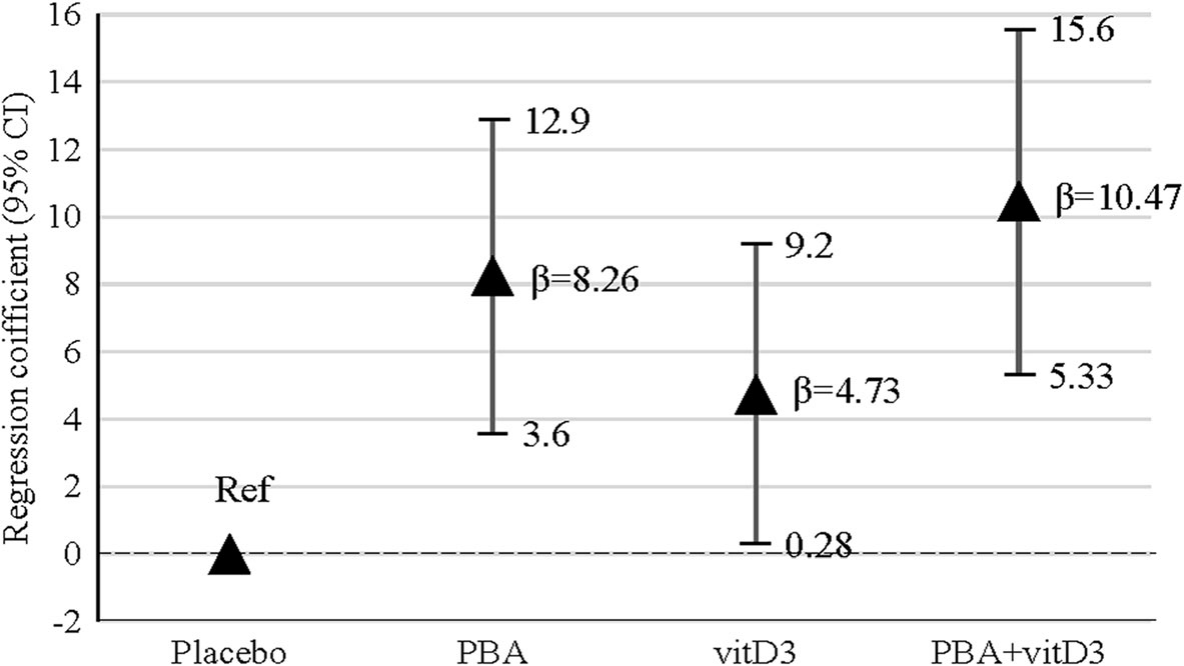
Generalized estimating equation (GEE) model was used to estimate the effect of adjunct therapy on LC3-expressing macrophages over time (week 0, 4, 8 and 12). β coefficient values are shown for three intervention groups (PBA, vitD_3_ and PBA + vitD_3_) compared to placebo. Arrowhead points represent the adjusted beta (β) coefficient values and vertical lines define 95% confidence intervals (CI). The analysis showed that mean % changes of LC3-expressing macrophage in PBA (β = 8.26; *p* < 0.001), vitD_3_ (β = 4.73; *p* = 0.037) and PBA+ vitD_3_ (β = 10.47; *p* < 0.001) were significantly higher compared to the placebo group

There was a basal number of LC3 expressing cells (4.3 ± 2.4) among the uninfected MDM in all the treatment arms of patients that did not change with different therapies or over the duration of the study period (Fig. 2b). In all groups a gradual increase in percentage of LC3-expressing H37Rv-infected MDM from week 0 up to week 12 was noted, indicating that the capacity of MDM to undergo autophagy increased with time during clinical recovery (Fig. 2a and b). Analysis by GEE model showed that mean changes of LC3-expressing macrophage percentage in PBA (β = 8.26, 95% CI = 3.61, 12.9, *p* < 0.001), vitD_3_ (β = 4.73, 95% CI = 0.28, 9.19, *p* = 0.037) and PBA+ vitD_3_ (β = 10.47, 95% CI = 5.33, 15.6, p < 0.001) were significantly higher compared to placebo (Fig. 3). Hence, oral adjunct therapy with PBA, vitD_3_ and the combined dose had a positive impact on priming for induction of autophagy in ex vivo-infected macrophages from TB patients that persisted up to additional 4 weeks after the adjunct therapy was completed.

### Endoplasmic reticulum stress markers and HBD1 in macrophages

**Fig. 4:**
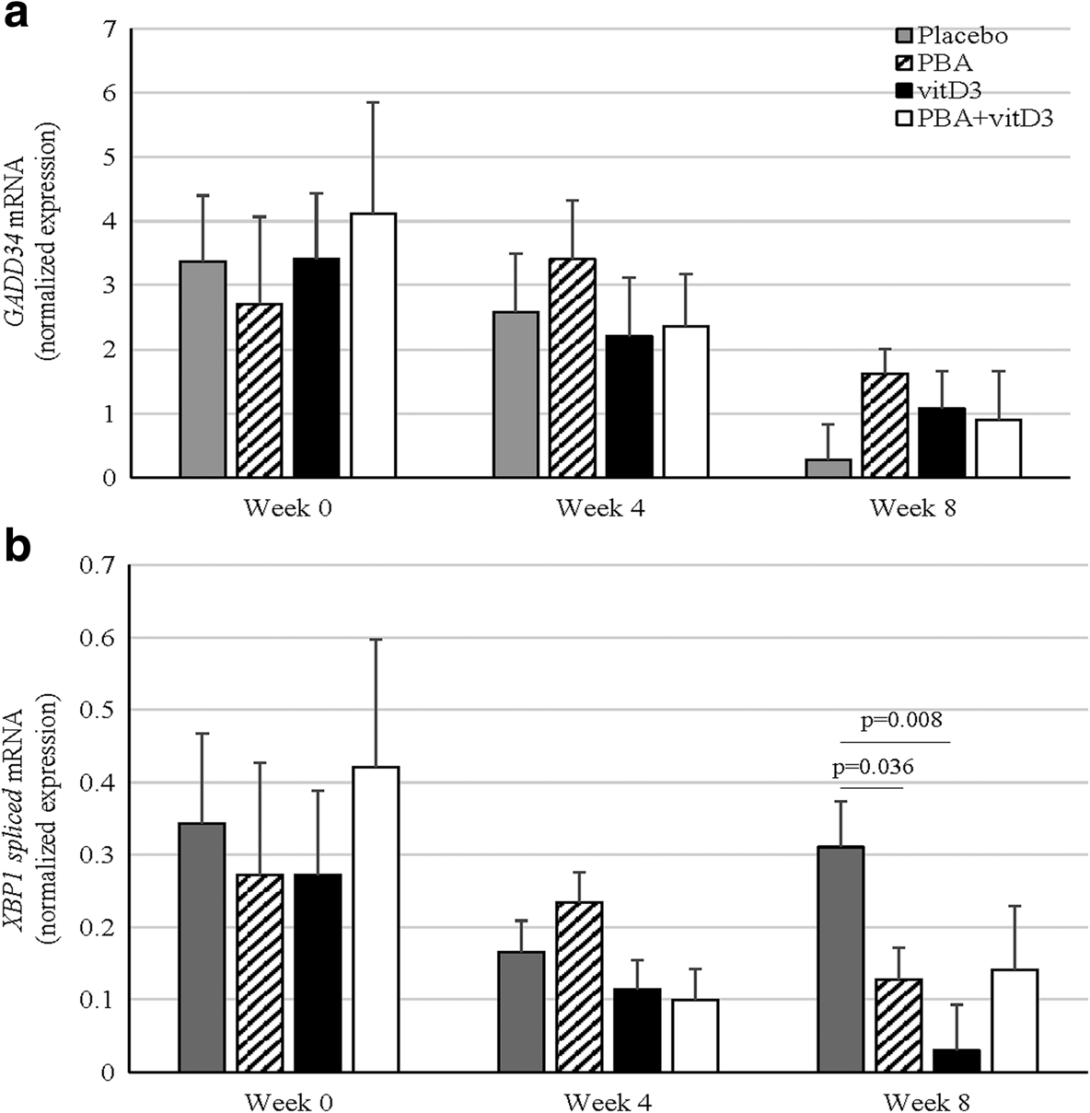
Real-time RT-PCR assays of *GADD34* and *XBP1spl* mRNAs from monocyte-derived-macrophages from TB patients receiving adjunct therapy with PBA and/or vitD_3_ or placebo (18/group) at week 0, week 4 and 8 after therapy. Data are normalized expression of *GAD34* (**a**) and *XBP1spl* (**b**) mRNAs presented as means ± standard deviation. The results showed that PBA and vitD_3_ groups showed a significant reduction in expression of ER stress gene *spliced XBP1* mRNA at week 8 compared to placebo

When MDM from TB patients were examined for ER stress genes, there were few time points where gene expression was below detection limit for *GADD34* or *XBP1spl*. These were considered as missing values in ANCOVA. All groups revealed a decline in expression of *GADD34* transcripts from week 0 to week 8. However, sex and age-adjusted ANCOVA analysis did not reveal any significant difference in the expression of *GADD34* transcripts between the treatment groups and placebo (Fig. 4a). Interestingly, a significant reduction in the expression of *XBP1spl* transcripts was found at week 8 in PBA (*p* = 0.036) and vitD_3_ (*p* = 0.008) groups compared to placebo but not in PBA + vitD_3_ (*p* = 0.12) group (Fig. 4b). Both PBA and vitamin D_3_ are known for their role in reducing ER stress in cells (26 or 27) [30]. Host directed therapy with PBA or vitamin D_3_ could individually dampen the ER stress in MDM, although the combined intervention did not reach a statistically significant level.

In addition to studying expression of cathelicidin LL-37 in MDM in the original study [25], we further assessed effect of PBA and/or vitD_3_ on induction of HBD1 in MDMs. The expression of HBD1 transcripts (2^–ΔΔC^T) in different intervention groups did not change significantly with time. Furthermore, the fold change in HBD1 transcripts did not exhibit any difference over time in any of the intervention groups compared to placebo (Table 3).

**Table 3:** Longitudinal change (week 0, 4, 8) in HBD1 in the treatment groups compared to Placebo (*n* = 18)

	Crude		Adjusted^a^	
HBD1	β(95% CI)	*p*-value	β(95% CI)	*p*-value
PBA (n = 18)	0.26 (−0.10, 0.62)	0.15	0.31 (−0.06, 0.67)	0.10
vitD_3_ (n = 18)	−0.05 (− 0.40, 0.29)	0.76	0.04 (− 0.31, 0.40)	0.81
PBA + vitD_3_ (n = 18)	0.21 (−0.15, 0.56)	0.25	0.28 (−0.09, 0.66)	0.14

Data is presented as beta coefficient with 95% confidence intervals in parentheses

*Abbreviations*: *CI* confidence interval, *HBD1* human beta-defenisn-1

^a^Adjusted for age, sex, duration of treatment, history of contact with active TB cases, time and the interaction between the treatment groups and time.

Statistical analysis was performed using generalized estimating equation (GEE) model. *P*-value of < 0.05 is significant

## Discussion

It is important to evaluate the role of immune-modulatory agents as host directed therapy in the treatment of TB that can improve efficacy of therapy, reduce or prevent detrimental outcomes of toxic drugs and improve patient compliance. Our interest in using PBA and/or vitD_3_ as HDT in the treatment of TB was based on their diverse roles in modifying the host defense system. Our findings showed that adjunctive treatment with PBA or vitD_3_ over 8 weeks’ time reduced concentration of cytokines/chemokines and endoplasmic reticulum stress in parallel to clinical recovery. All three adjunctive therapies increased frequency of autophagy in macrophages compared to placebo.

TB is a chronic infectious disease with a spectrum of symptoms and presentations, wherein cytokines and chemokines play important roles in manipulation of the immune responses, containment of *Mtb*, pathogenesis and disease manifestations. Various studies have compared serum cytokine/chemokine profiles of patients with different clinical presentations of TB, to establish the expression levels as early surrogate biomarkers of therapeutic responses, bacteriological confirmation of TB and sputum culture conversion [31–33]. We measured cytokines and chemokines in culture supernatants of PBMC instead of serum or plasma, and the findings were similar to previous studies, showing marked decline of serum cytokine/chemokine concentrations at different treatment stages [14, 33–37]. Particularly we found that compared to conventional anti-TB treatment in the placebo group, cytokine/chemokine reduction was greater in the PBA or vitD_3_ groups that occurred in parallel to clinical recovery [25] (reduced TB score), suggesting a role of PBA and vitD_3_ in dampening of inflammatory responses.

Mycobacterial infection has been shown to induce ER stress and damage ER. Cells deal with ER stress by activation of the unfolded protein response (UPR), but chronic activation of these pathways can eventually result in apoptosis and lung injury [18, 38–40]. Several biomarkers of ER stress have been used in various studies by assessing activation of the UPR; spliced X-box binding protein-1 (*XBP1spl*) mRNA has been shown to be a reliable marker in revealing dose-dependent changes in the transcript level of this gene [28]. Mycobacterial secreted antigens such as ESAT-6 has been shown to increase *XBP1spl* as well as other ER stress genes in epithelial cell lines [38]. Toll-like receptor activation was also shown to increase splicing of the transcription factor *XBP1* in the absence of other signs of activation of the UPR, and this was found to regulate innate immune responses in macrophages [41]. PBA is a classic ER stress inhibitor; it is a chemical chaperone that improves ER folding capacity and trafficking of mutant proteins out of the ER, stabilization of protein conformation and thereby reduces accumulation of misfolded proteins in ER lumen. In cell lines, experimental models and tissue samples, PBA has been shown to reduce expression of GRP-78, CHOP and other UPR-related markers such as *ATF6, XBP1* and phosphorylation of *eIF2α* [27, 42–44]. Vitamin D is also considered a natural ER stress reliever [30, 45]. Down-regulation of *GADD34* transcripts after 8 weeks of therapy was evident in all 4 arms, however down-regulation of *spliced XBP1* was evident only in PBA and vitD_3_ arms compared to placebo. Thus, in vivo effect of PBA and vitD_3_ adjunct treatments was demonstrated in diminishing ER stress in MDM during TB infection that may have a role in improving clinical outcome. Moreover, diminished inflammatory cytokine responses as we have shown here may be linked to suppression of ER stress [46, 47].

Autophagy is an important physiological process that is applied by macrophages to control and eliminate intracellular pathogens. We have earlier demonstrated in a mechanistic study that PBA, active vitD_3_ separately and in combination induced autophagy in macrophages from healthy donors when infected in vitro with *Mtb* [23], increased the intracellular killing of *Mtb* compared to untreated MDM and induced LL-37 in cell supernatants of macrophages and lymphocytes after oral ingestion by healthy adults [22]. The novel activity of PBA as an inducer of autophagy was LL-37-dependent. We further showed in the present study that, all three adjunctive therapies (PBA, vitD_3_ and PBA + vitD_3_) in the clinical trial [25] promoted autophagy in ex vivo infected macrophages from the patients, indicating that the inducers can prime the macrophages for autophagy response. Moreover, the autophagy process occurred in parallel to induction of LL-37 in macrophage/PBMC [25], continuing up to an additional week after the adjunct therapy was completed. MDM-mediated elimination of *Mtb* in the vitamin D and PBA + vitamin D_3_ arms in the trial thus strongly suggest a role of the autophagy process in the eradication. Autophagy also plays a housekeeping role in removing misfolded, unfolded or aggregated proteins and clearing damaged organelles, such as ER and mitochondria. In view of the involvement of the UPR in activating autophagy, it is interesting to note that whereas treatment increased autophagy, it appeared to decrease activation of the UPR to ER stress. This suggests that mechanisms distinct from the UPR are involved in the observed treatment-induced autophagy.

Human beta defensin-1 (HBD1) is produced constitutively by all human epithelia and some immune cells. Monocyte-derived-macrophages and -dendritic cells both express HBD1 mRNA, showing increased expression after activation with IFN-γ and/or lipopolysaccharide in a dose- and time-dependent fashion [48]. When expression of HBD1 transcripts was studied in MDMs from the patients we did not observe any increase or down-regulation of the peptide in the treatment groups compared to placebo during the course of TB disease. Thus, PBA or vitD_3_ did not seem to have any modulating effect on HBD1 expression in peripheral MDM as seen with LL-37 [25].

One of the limitations of this study was the small size used per treatment group for evaluation of ER stress genes and HBD1 genes. The expression of transcripts was studied only in macrophages, not other cells in PBMC. This was done because of unavailability of adequate number of matched/paired samples from different time points and suboptimum concentrations of specimens remaining from the clinical trial. Our analysis of activation of the UPR was restricted to measurement of *GADD34* and *XBP1spl* because of limitations in the availability of RNA, and therefore other UPR markers (including protein markers) should be assessed in future studies. Only one autophagy marker LC3 was used instead of a combination of markers such as *ATG 5* and *Beclin-1*; however, our earlier studies showed the LC3 marker as reliable for assessment of autophagy in macrophages [23]. The combined adjunct therapy of PBA and vitD_3_ did not exhibit any effect on cytokines/chemokines or ER stress reduction in TB patients and require further investigations. Notably, the combined treatment with PBA and vitD_3_ of the patients exhibited the fastest sputum culture conversion [25]. Another limitation was that we measured cytokines and chemokines in saponin-treated cell culture fluid instead of serum or plasma as traditionally done [10–14, 31–33]. Measurement of cytokines in the serum does not accurately reflect active secretion of cytokines due to an ongoing infection; moreover it is affected by clearance from the circulation. Our hypothesis is that the PBMC from TB patients are in vivo activated and they do not require ex-vivo stimulation; release of cytokines/chemokines from unstimulated cells into the culture supernatant indicate active secretion and thus are more relevant to the ongoing disease process. However, use of saponin allowed excretion of intra-cellular cytokines into the culture supernatant which was not a spontaneous release of cytokines.

## Conclusions

demonstration of clinically beneficial immunomodulation that occur in parallel to improved treatment outcomes suggest that use of repurposed agents such as Phenylbutyrate and vitamin D_3_ can be a valuable strategy in HDT against drug-sensitive TB. The toxicities and poor treatment outcomes of current therapies against MDR-TB necessitate newer approaches to improve the management and control of MDR-TB. Development of host-directed therapies integrated with *Mtb*-targeted chemotherapy provides a complicated challenge because temporal events of infection and host immunity may play a critical role in determining HDT efficacy. Thus, the therapeutic potential of PBA and vitD_3_ HDT against multidrug resistant TB warrants urgent clinical evaluation in well-designed multi-center clinical trials in endemic settings.

## Additional file

Additional file 1: **Table S1.** Descriptive statistics of the current studied patients and the patients who were not studied. **Table S2.** Longitudinal change (week 0, 4, 8 and 12) in TB score in the intervention groups compared to placebo. **Table S3.** Concentrations of inflammatory cytokines and chemokines in treatment groups at different time intervals. (DOCX 24 kb)

## Abbreviations

95% CI: 95% confidence interval
AMPs: Antimicrobial peptides
DOTS: Directly observed therapy short-course
ER: Endoplasmic-reticulum
GADD34: Growth arrest and DNA damage inducible protein 34
GEE: Generalized estimating equation
HBD1: Human β-defensin-1
HDT: Host-directed therapies
MDM: Monocyte-derived-macrophages
*Mtb*: *Mycobacterium tuberculosis*
PBA: Phenylbutyrate
PBMC: Peripheral blood mononuclear cells
TB: Tuberculosis
vitD_3_: Vitamin D_3_
*XBP1spl*: Spliced X-box binding protein-1
