# Supplementary material for: Immune responses in the treatment of drug-sensitive pulmonary tuberculosis with phenylbutyrate and vitamin D3 as host directed therapy
Source: BMC Infect Dis. 2018 Jul 4;18:303. doi: 10.1186/s12879-018-3203-9 (PMC6033279; doi:10.1186/s12879-018-3203-9)
Supplement: Supplementary file 1 — Table S1. Descriptive statistics of the current studied patients and the patients who were not studied. Table S2. Longitudinal change (week 0, 4, 8 and 12) in TB score in the intervention groups compared to placebo. Table S3. Concentrations of inflammatory cytokines and chemokines in treatment groups at different time intervals. (DOCX 24 kb) [file 12879_2018_3203_MOESM1_ESM.docx]

Immune responses in the treatment of drug-sensitive pulmonary tuberculosis with phenylbutyrate and vitamin D_3_ as host directed therapy

*Rokeya Sultana Rekha^1,2^, *Akhirunnessa Mily^1^, Tajnin Sultana^1^, Ahsanul Haq^1^, Sultan Ahmed^2^, S. M. Mostafa Kamal^3^, Annemarie van Schadewijk^4^, Pieter S. Hiemstra^4^, Gudmundur H. Gudmundsson^5^, ^§^Birgitta Agerberth^2^, ^§^Rubhana Raqib^1^

^1^Immunobiology, Nutrition and Toxicology Laboratory, Infectious Diseases Division, icddr,b, Dhaka, Bangladesh; ^2^Department of Laboratory Medicine, Clinical Microbiology, Karolinska Institutet, Karolinska University Hospital, Stockholm, Sweden; ^3^National Institute of the Diseases of the Chest and Hospital, Mohakhali, Dhaka, Bangladesh; ^4^Department of Pulmonology, Leiden University Medical Centre, Leiden, the Netherlands; ^5^Biomedical Center, University of Iceland, Reykjavik, Iceland.

*These authors contributed equally to this work as first authors

^§^These authors contributed equally to this work as senior authors

**Short title**: cytokines in host-directed therapy of TB

**Key words**: *Mycobacterium tuberculosis*; cytokines, chemokines; endoplasmic reticular stress, human beta-defensin-1 (HBD1)

**Correspondence**:

Dr. Rubhana Raqib,

Infectious Disease Division, icddr,b,

68 Shaheed Tajuddin Ahmed Sarani, Dhaka-1212, Bangladesh.

Tel.: +8802-9827068; Fax: +8802-8812529;

E-mail: [rubhana@icddrb.org](mailto:rubhana@icddrb.org)

**Table S1:** Descriptive statistics of the current studied patients and the patients who were not studied.

|  |  | Placebo  (n^1^=32 & n^2^=32) | | PBA  (n^1^=26 & n^2^=32) | | vitD_3_  (n^1^=31 & n^2^=31) | | PBA+vitD_3_  (n^1^=33 & n^2^=32) | |
| --- | --- | --- | --- | --- | --- | --- | --- | --- | --- |
| Variables |  | Mean | p | Mean | p | Mean | p | Mean | p |
| ^a^Age, years | Not included | 26.41±7.31 | 0.63 | 26.92±8.09 | 0.69 | 29.10±9.31 | 0.10 | 26.70±5.51 | 0.79 |
|  | Current | 27.38±8.65 |  | 27.72±7.25 |  | 25.42±8.15 |  | 27.16±8.38 |  |
| ^b^Male | Not included | 19 (48.7%) | 0.79 | 15 (40.5%) | 0.38 | 18 (46.2%) | 0.43 | 21(48.8%) | 0.66 |
|  | Current | 20 (51.3%) |  | 22 (59.5%) |  | 21 (53.8%) |  | 22 (51.2%) |  |
| ^b^History of contacts | Not included | 9 (47.4%) | 0.78 | 5 (31.3%) | 0.19 | 9 (60.0%) | 0.37 | 13 (61.9%) | 0.21 |
|  | Current | 10 (52.6%) |  | 11 (68.8%) |  | 6 (40.0%) |  | 8 (38.1%) |  |
| ^b^BCG given | Not included | 19(46.3%) | 0.43 | 16 (45.7%) | 0.86 | 23 (53.1%) | 0.34 | 23 (50.0%) | 0.84 |
|  | Current | 22 (53.7%) |  | 19 (54.3%) |  | 26 (47.3%) |  | 23 (50.0%) |  |
| ^a^Weight, Kg | Not included | 42.32±6.34 | 0.18 | 41.94±8.56 | 0.10 | 44.30±8.93 | 0.53 | 44.21±7.75 | 0.91 |
|  | Current | 44.75±8.20 |  | 45.74±8.91 |  | 42.91±8.70 |  | 44.00±7.89 |  |
| ^a^Duration of illness, days | Not included | 55.81±25.52 | 0.28 | 50.58±19.04 | 0.99 | 53.87±27.29 | 0.51 | 49.27±25.98 | 0.63 |
|  | Current | 48.91±25.42 |  | 50.63±21.80 |  | 58.39±16.81 |  | 52.47±27.58 |  |
| ^b^sputum smear |  |  |  |  |  |  |  |  |  |
| >3 AFB | Not included | 11 (42.3%) | 0.30 | 8 (33.3%) | 0.13 | 18 (52.9%) | 0.61 | 16 (51.6%) | 0.89 |
|  | Current | 15 (57.7%) |  | 16 (66.7%) |  | 16 (47.1%) |  | 15 (48.4%) |  |

Abbreviations: BCG, *Bacillus Calmette–Guérin;* AFB, acid-fast bacilli;

Data is presented as mean ± standard deviation or number with percentage in parentheses

n^1^ patients who were not studied

n^2^ current studied patients

^a^p values obtained using independent sample t-test

^b^p values obtained using χ^2^-test

**Table S2.** Longitudinal change (week 0, 4, 8 and 12) in TB score in the intervention groups compared to placebo.

|  | Original, n=249 | |  | Current, n=127 | |
| --- | --- | --- | --- | --- | --- |
|  | Β (95% CI) | p-value |  | Β (95% CI) | p-value |
| TB score |  |  |  |  |  |
| PBA | -0.49 (-0.82, -0.16) | 0.004 |  | -0.56 (-1.02, -0.09) | 0.01 |
| vitD_3_ | -0.15 (-0.48, 0.18) | 0.37 |  | 0.17 (-0.32, 0.65) | 0.50 |
| PBA+vitD_3_ | -0.24 (-0.56, 0.09) | 0.15 |  | -0.04 (-0.51, 0.43) | 0.87 |

Abbreviations: PBA, phenylbutyrate; vitD_3_, vitamin D_3_

Data is presented as beta coefficient with 95% confidence intervals in parentheses.

^a^Adjusted for age, sex, duration of treatment, history of contact with active TB cases, time and the interaction between the treatment groups and time.

Statistical analysis was performed using generalized estimating equation (GEE) model. P-value of <0.05 is significant.

**Table S3**. Concentration of inflammatory cytokines and chemokines in treatment groups at different time intervals.

| Cytokines/ |  | Placebo (n=32) | PBA (n=32) | vitD_3_ (n=31) | PBA+vitD_3_ (n=32) |
| --- | --- | --- | --- | --- | --- |
| chemokines | Time point |  |  |  |  |
| TNF-α | Baseline | 120  (30, 19300) | 110  (1, 1090)* | 130  (10, 1910) | 110  (20, 3389200) |
|  | Week-8 | 160  (40, 37000) | 100  (20, 57800) | 130  (20, 13500) | 109  (30, 260200) |
| IL-10 | Baseline | 30  (10, 8380) | 40  (0.1, 500) | 30  (4, 2320) | 40  (10, 2000) |
|  | Week-8 | 29  (1, 5490) | 20  (4, 2000) | 25  (1, 1130) | 50  (0.1, 4690 ) |
| IL-8 | Baseline | 68890  (3040, 3200000) | 35310  (1010, 3200000) | 87630  (8700, 3200000) | 62820  (3030, 3200000) |
|  | Week-8 | 59830  (7000, 3200000) | 45300  (2010, 3200000) | 32600  (8690, 3200000) | 41620  (2820, 3200000) |
| GM-CSF | Baseline | 100  (10, 450) | 100  (0.1, 550) | 110  (20, 700) | 100  (0.1, 480) |
|  | Week-8 | 90  (40, 470) | 60  (40, 530) | 70  (20, 420) | 80  (0.1, 500) |
| IL-17 | Baseline | 50  (10, 410) | 70  (0.1, 400)* | 40  (10, 300) | 40  (10, 330) |
|  | Week-8 | 60  (10, 430) | 40  (20, 330) | 60  (10, 300) | 40  (10, 220) |
| CCL11 | Baseline | 30  (10, 100) | 30  (10, 100)* | 30  (1, 60) | 20  (2, 40) |
| (Eotaxin) | Week-8 | 30  (2, 110) | 20  (4, 50) | 30  (10, 100) | 20  (10, 90) |
| CCL5 | Baseline | 2450  (1670, 3640) | 2320  (1010, 3030) | 2180  (1000, 4100) | 2160  (1200, 2940) |
| (RANTES) | Week-8 | 2110  (1320, 3440) | 2100  (540, 3190) | 2520  (650, 1710) | 2000  (1000, 2810) |
| CXCL10 | Baseline | 1010  (230, 18450) | 700  (100, 7700) | 930  (40, 8000) | 550  (100, 5100) |
| (IP-10) | Week-8 | 1080  (10, 34900) | 500  (70, 13800) | 720  (70, 7570) | 740  (140, 9540) |
| FGF-Basic | Baseline | 40  (1, 120) | 50  (1, 80) | 50  (1, 80) | 30  (1, 150)* |
|  | Week-8 | 40  (1, 100) | 50(1, 90) | 30  (1, 130) | 20  (1, 80) |
| PDGF- β | Baseline | 1880  (920, 7130) | 1450  (320, 5690) | 1480  (370, 2730) | 1490  (510, 3440)* |
|  | Week-8 | 1280  (340, 4090) | 1160  (370, 4000) | 870  (160, 3230) | 620  (280, 4170) |

Data are expressed as median with minimum and maximum values in brackets. Concentration of cytokines and chemokines are given in pg/ml. P value between baseline and week 8 was calculated using Wilcoxon Signed Ranks test. *p<0.05 is considered significant.
